# T-Cell Responses after Rotavirus Infection or Vaccination in Children: A Systematic Review

**DOI:** 10.3390/v14030459

**Published:** 2022-02-23

**Authors:** Natasha Makabilo Laban, Martin Rhys Goodier, Samuel Bosomprah, Michelo Simuyandi, Caroline Chisenga, Obvious Nchimunya Chilyabanyama, Roma Chilengi

**Affiliations:** 1 Department of Infection Biology, Faculty of Infectious and Tropical Diseases, London School of Hygiene and Tropical Medicine, London WC1E 7HT, UK; martin.goodier@lshtm.ac.uk; 2 Enteric Disease and Vaccine Research Unit, Centre for Infectious Disease Research in Zambia, Lusaka P.O. Box 34681, Zambia; samuel.bosomprah@cidrz.org (S.B.); michelo.simuyandi@cidrz.org (M.S.); caroline.chisenga@cidrz.org (C.C.); obvious.chilya@cidrz.org (O.N.C.); roma.chilengi@cidrz.org (R.C.); 3 Flow Cytometry and Immunology Facility, Medical Research Council Unit, The Gambia at London School of Hygiene and Tropical Medicine, Fajara, Banjul P.O. Box 273, The Gambia; 4 Department of Biostatistics, School of Public Health, University of Ghana, Accra P.O. Box LG13, Ghana

**Keywords:** T-cell, rotavirus, child, infection, vaccination

## Abstract

Cellular immunity against rotavirus in children is incompletely understood. This review describes the current understanding of T-cell immunity to rotavirus in children. A systematic literature search was conducted in Embase, MEDLINE, Web of Science, and Global Health databases using a combination of “t-cell”, “rotavirus” and “child” keywords to extract data from relevant articles published from January 1973 to March 2020. Only seventeen articles were identified. Rotavirus-specific T-cell immunity in children develops and broadens reactivity with increasing age. Whilst occurring in close association with antibody responses, T-cell responses are more transient but can occur in absence of detectable antibody responses. Rotavirus-induced T-cell immunity is largely of the gut homing phenotype and predominantly involves Th1 and cytotoxic subsets that may be influenced by IL-10 Tregs. However, rotavirus-specific T-cell responses in children are generally of low frequencies in peripheral blood and are limited in comparison to other infecting pathogens and in adults. The available research reviewed here characterizes the T-cell immune response in children. There is a need for further research investigating the protective associations of rotavirus-specific T-cell responses against infection or vaccination and the standardization of rotavirus-specific T-cells assays in children.

## 1. Introduction

Rotavirus is the leading cause of life-threatening diarrhea among young children, particularly in those below five years of age [1,2]. Globally, rotavirus has been responsible for approximately 258 million diarrhea episodes and an estimated 128,515 diarrhea deaths in this population with the largest burden within Sub-Saharan Africa [3]. Fortunately, rotavirus vaccines are widely available and have significantly contributed to reductions in rotavirus-associated diarrhea morbidity and mortality globally [4,5,6]. However, despite being discovered nearly half a century ago in 1973 and more than a decade since vaccine introduction, immune mechanisms, and correlates of protection against rotavirus remain poorly understood [7].

In humans, rotavirus is transmitted via a fecal-oral route and is known to predominantly infect and replicate in mature enterocytes of the intestinal epithelium inducing innate and adaptive humoral and cellular immune responses [8]. In children, repeated rotavirus infection leads to a lower likelihood of subsequent rotavirus infections and reduced occurrence of moderate to severe diarrheal disease suggesting the development of immune memory [9]. This acquired, non-sterilizing immunity is derived from a combination of gut secretory and humoral antibody and cell-mediated immune effectors with neutralizing antibodies directed against the viral capsid proteins and viral epitope recognition by T-cells thought to play an important role in protection [8]. However, immune parameters correlating with protection against rotavirus in humans are yet to be demonstrated [10].

Rotavirus-specific antibodies are well documented and frequently studied in children as immune markers of previous infection or vaccination [11]. However, even though they are recognized as being important for protection, it is generally appreciated that these antibody markers are sub-optimal correlates of protection [12,13]. In contrast, there is sparse data on the underlying T-cell immune responses to rotavirus infection or vaccination, particularly in children, and even fewer still have studied the role of this T-cell immunity in protection against rotavirus. The current understanding of rotavirus T-cell mediated immunity has for the most part been achieved through studies in animal models which have shown that T-cells have crucial roles in suppression of rotavirus replication, clearance of infection, and generation of antibody responses associated with protection [10,14,15,16].

As rotavirus remains a cause of high morbidity and mortality in children, especially in the developing world [3], it is necessary to fully understand the immune mechanisms underlying protection. Improved knowledge on T-cell-mediated rotavirus immunity can inform vaccine development and is particularly important considering the sub-optimal antibody immune correlates and the consistent observation of markedly lower vaccine immunogenicity and efficacy in children within high rotavirus burden regions [17]. We, therefore, conducted a systematic review of literature on T-cell responses to rotavirus in children to consolidate currently available knowledge on the characteristics of T-cell immunity to rotavirus in this population including its association with the antibody responses.

## 2. Materials and Methods

### 2.1. Literature Search Strategy

We followed the Preferred Reporting Items for Systematic Reviews and Meta-Analysis (PRISMA) checklist (Table S1) in the preparation of the systematic review manuscript [18]. The literature search was conducted in Embase (1947 to March 2020), MEDLINE (1946 to March 2020), Web of Science (1970 to 2020), and Global Health (1910 to week 9 2020) electronic databases using a combination of “T-cell”, “rotavirus” and “child” keywords to identify relevant articles (File S1).

### 2.2. Inclusion Criteria

Studies included in this review were those that were primary research, were conducted among children or used child-derived samples in any region of the world, reported T-cell immune responses to rotavirus, had full English text available and had rotavirus as the main focus of the study. There was no restriction to study design, but we restricted selection to articles published after 1973, the year rotavirus was discovered.

### 2.3. Exclusion Criteria

We excluded studies that did not include children or child-derived samples, did not report T-cell responses against rotavirus, or had no English full text available. Non-primary research including review articles and conference abstracts were also excluded.

### 2.4. T-Cell Responses

The T-cell responses considered in the systematic review were T-cell quantity (counts, ratios, frequencies), phenotype (activation, cell surface markers, epitopes) function (cytokine secretion), activity (proliferation), and kinetics (pre and post-infection or vaccination, durability) for all CD4 and CD8 T-cells subsets.

### 2.5. Study Selection and Data Extraction

EndNote reference manager software was used to remove duplicate articles identified from the search strategy. The resulting unique articles were imported into Rayyan web-tool software for additional duplicate identification and article selection. Three reviewers (NML, CC, MS) independently selected potentially eligible articles by screening the title and abstract of all unique articles for the keywords using the Rayyan web-tool software. Full texts of articles selected by all three reviewers combined were retrieved and assessed for eligibility using the inclusion and exclusion criteria. Articles concordantly selected as eligible by the three reviewers were included in the review and those concordantly rejected were excluded from the review. Discordance in selection was discussed and re-assessed together by all three reviewers until a consensus on inclusion or exclusion was made. Data were extracted into an excel sheet to capture information on the author, year of publication, study location, study design, characteristics of the child population, sample size, rotavirus context (rotavirus infection or vaccination), T-cell responses, and laboratory methods used for measures of T-cell immunity.

### 2.6. Quality Assessment and Data Synthesis

We reviewed published articles of similar nature to our systematic review to identify potential appraisal tools and we adapted a recently published quality assessment tool [19] and quality level thresholds (0% to 39% = low, 40% to 69% = moderate, and 70% to 100% = high) [20] for our critical appraisal (Table S2). One author (NML) conducted the quality assessment which was reviewed by two other authors (SB and ONC). Due to the wide heterogeneity in laboratory methodology and reported T-cell response across the studies included in the systematic review, formal quantitative meta-analysis was not conducted, and results were presented in a thematic narrative format.

## 3. Results

### 3.1. Literature Search Results

**Figure 1:**
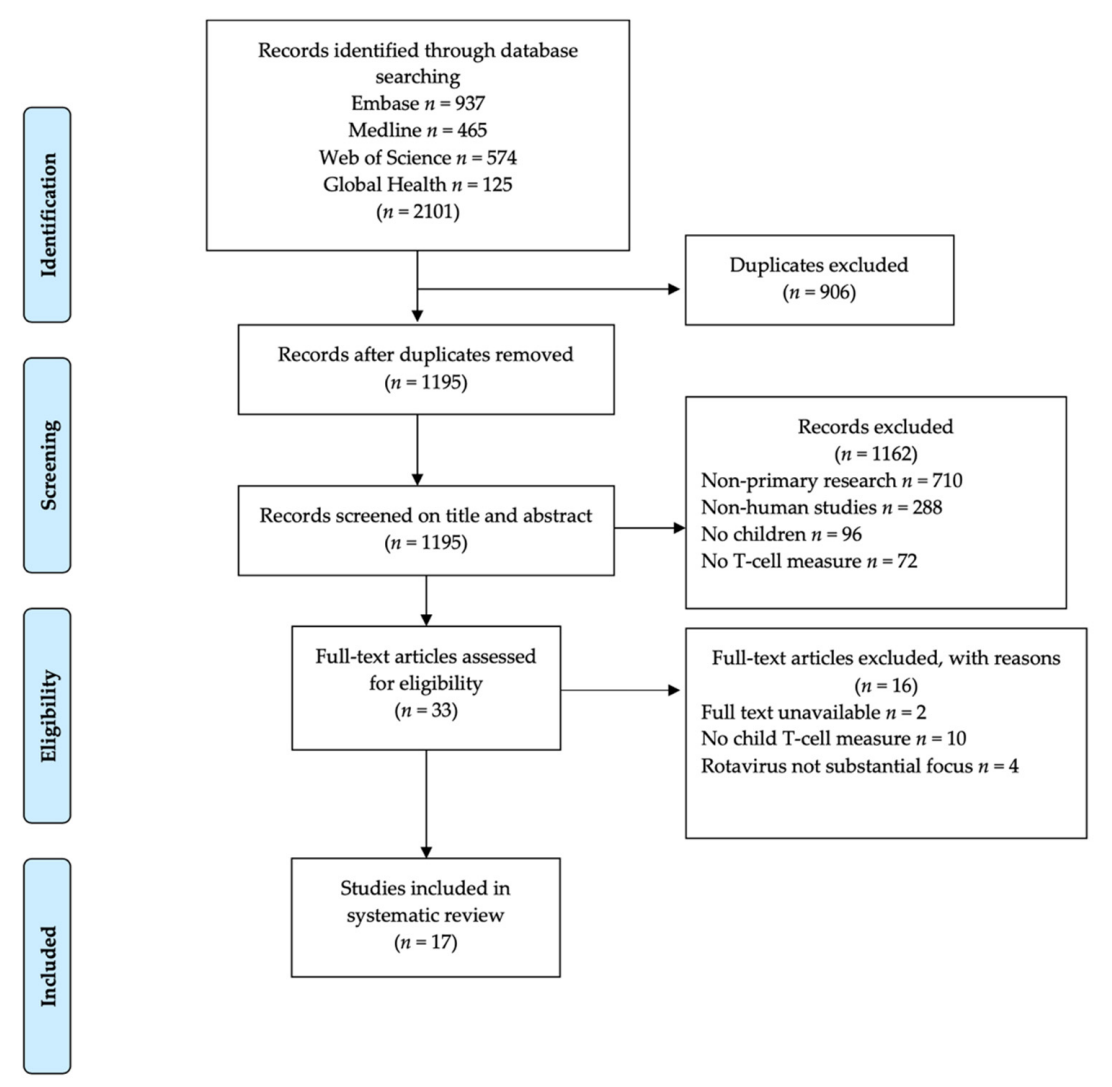
Flow chart of literature search results and article selection process.

Articles retrieved from the literature search comprised 937 from Embase, 465 from MEDLINE, 574 from Web of Science, and 125 from Global Health electronic databases giving a total of 2101 articles identified. After the removal of 906 duplicate articles, a resulting total of 1195 articles were screened for eligibility based on title and abstract and an additional 1162 articles were excluded because they were non-primary research (*n* = 710), were not about rotavirus in humans (*n* = 288,) did not include children (*n* = 96), did not report T-cell responses (*n* = 72). The remaining 33 articles underwent further screening for eligibility by full text based on set inclusion criteria. After full-text screening, a further 16 articles were excluded because they did not have full text available to the reviewers (*n* = 2), did not report T-cell responses for children (*n* = 10), and rotavirus was not the main focus (*n* = 4). This resulted in 17 articles that met the inclusion criteria and were included in the systematic review as summarized in Figure 1.

### 3.2. Characteristics of Articles Included in Systematic Review

Among the seventeen studies included in the systematic review, the earliest study identified was published in 1988 and the latest in 2018. Most of the studies were conducted among children in the Americas (9/17) followed equally by Europe (3/17) and Asia (3/17) while the least number of studies (2/17) was conducted among African children. Ten studies reported T-cell immunity in the context of rotavirus infection, two studies reported T-cell responses to rotavirus vaccination, and five studies reported rotavirus-specific T-cell response in healthy children. Laboratory methods used to measure T-cell responses varied across studies and included flow cytometry, lymphoproliferation, microscopy, indirect fluorescence microscopy, gene microarray, and enzyme-linked immunospot (ELISpot) assays. Different types of T-cell outcomes in response to mitogen, human rotavirus, and non-human rotavirus antigens were reported across studies. More detailed characteristics of the studies included in the systematic review are as outlined in Table 1.

### 3.3. Quality Assessment of Individual Studies

Of the included studies, 15/17 (88.2%) were observational studies while only 2/17 (11.8%) made use of experimental designs in the form of randomized controlled trials (Table 1). Using our adapted appraisal tool and threshold definitions of study quality, most articles were of moderate quality 11/17 (65%). The remaining 6/17 (35%) articles were appraised as high-quality articles of which the majority 5/6 (83%) were published in more recent years (Table 1). Most studies included in the review provided adequate information on research gaps around immunity to rotavirus, including research questions and rationales for the study of T-cell-specific responses to rotavirus. However, there was generally poor methodological reporting for most studies with minimal to no detailed information provided on the exact study design employed, calculations, and assumptions for stated samples sizes or specification of inclusion and exclusion criteria for children or child-derived samples included in the studies. In most studies, there was also a poor presentation of participant or sample flow from recruitment to laboratory testing results as well as little to no information on children’s background characteristics (Table S2).

### 3.4. T-Cell Proliferation against Rotavirus Develops and Broadens Reactivity with Increasing Age

Children can mount detectable T-cell proliferation to different strains of rotavirus after in-vitro stimulation which is associated with age. As shown in Table 2, six studies reported induction of T-cell proliferation against human and non-human rotavirus strains and its relationship with the child’s age. Children with acute rotavirus diarrhea had more positive and significantly higher T-cell proliferation to rotavirus antigen compared to healthy children. Among healthy children, T-cell proliferation was absent in newborns, minimally present in children aged <6 months but became more commonly detected in older age groups of children [22,25,26,28,37]. In contrast to this, however, one study also reported evidence of detectable T-cell proliferation in newborn children [28]. In healthy children, although T-cell proliferation to a human rotavirus strain was observed to be stronger than that against a bovine rotavirus strain, a positive correlation of T-cell reactivity was observed between the strains [26]. By the age of 2 years old and beyond, most children had developed T-cell reactivity against two strains of human rotavirus and against rhesus rotavirus strains [28]. However, T-cell proliferation against two different human rotavirus strains has also been observed among children aged <2 years old with acute and convalescent rotavirus diarrhea caused by different infecting rotavirus strains [29].

### 3.5. Rotavirus T-Cell Proliferation and Frequency Coincides with Antibody Responses but Is More Transient

Six studies reported T-cell immunity in association with rotavirus antibody responses as shown in Table 3. T-cell responses were observed more frequently in rotavirus antibody seropositive than seronegative children and among secondary than primary infections indicating that both memory T-cell and antibody responses are induced by rotavirus exposure and built from repeated exposure [25,26,31]. The strength and magnitude of T-cell responses occurred in very close association with the antibody response. Makela et al. showed that generally, lower antibody titers to rotavirus were accompanied by minimal or absent T-cell responses while increased antibody responses were associated with stronger T-cell responses. However, strong T-cell immunity was also observed in the absence of increasing antibody responses in a single child in this study and although firm conclusions cannot be made based on this lone observation, it highlights the need to detect both antibody and cellular responses in assessing rotavirus immunity [26]. Compared to antibodies that persisted long after infection, T-cell responses were more transient, detectable two to eight weeks and three to five months post-infection but declining as early as 5 months to nearly undetectable levels within 12 months post rotavirus exposure [26,29]. However, both T-cell and antibody responses were minimal during acute rotavirus infection but more frequent during convalescence [29]. Unlike antibodies present at birth, T-cell immunity was generally absent in early infancy (<6 months) developing much later in infancy and may therefore be a better indicator of active infant immunity than antibodies and distinguish from passive maternal immunity in the very young infants [28]. Both T-cell and antibody responses can be mounted against different infecting rotavirus strains indicating an inability to clearly distinguish rotavirus P and G serotypes [29]. Rotavirus-specific CD4 T-cells are positively associated with antibody responses, while regulatory T-cells may either have a positive or negative association with the antibody response to rotavirus [35]. One study among T-cell deficient children further emphasized intimate associations between T-cell immunity and antibody response in the context of clearance of rotavirus infection. Wood et al. described chronic rotavirus infection in two children with congenital T-cell deficiency [36]. In a child with cartilage hair hypoplasia associated T-cell deficiency and acute rotavirus diarrhea, no serum antibody immune response to rotavirus was detected. Likewise, no significant proliferative response to rotavirus was observed ~1 year after the onset of diarrhea and diarrhea persisted over an 18-month period characterized by poor weight gain and failure for the child to thrive despite treatment. In the same study, a second child with CHARGE congenital abnormalities and DiGeorge syndrome associated T-cell deficiency who was infected with rotavirus, the rotavirus IgG antibody response was undetectable two months after rotavirus infection and despite treatment, this child failed to thrive and died at 5 months old.

### 3.6. CD4 and CD8 T-Cells Are of Low Circulating Frequency in Acute Rotavirus

Five studies reported a lower circulating frequency of CD4^+^ and CD8^+^ T-cells in response to acute rotavirus infection. In one study, while healthy children had normal proportions of CD3^+^, CD4^+^, and CD8^+^ T-cell subsets, children with acute rotavirus diarrhea had selectively lowered CD4^+^ T-cell proportion and a low CD4^+^:CD8^+^ T-cell ratio [22]. A case study of a single child with rotavirus diarrhea showed a depressed CD4^+^ T-cell frequency and CD4^+^:CD8^+^ ratio in an acute phase that persisted up to one-month post-infection but normalized by convalescent period [23]. In another two studies close to half of the children with rotavirus diarrhea had absolute lymphopenia compared to children with or without previous rotavirus exposure but with non-rotavirus diarrhea and the majority of children with acute (<7 days after the onset of illness) rotavirus diarrhea had total whole blood lymphocyte counts less than the lower limit of the normal count range in healthy children [27,34]. Additionally, among children with previous rotavirus exposure and those with rotavirus diarrhea, few had detectable cytokine-producing rotavirus-specific CD4 or CD8 T-cells [27]. Likewise, flow cytometry and gene expression T-cell analysis of children with rotavirus diarrhea revealed significantly lower mean frequencies of CD4^+^ and αβ^+^CD4^+^ T-cells, CD8^+^ and αβ^+^CD8^+^ T-cells and T-cell associated gene expression in children with rotavirus diarrhea in the acute phase than in healthy controls. In the convalescent phase, however, the frequencies of these T-cell populations significantly increased to similar levels observed in healthy children. Exceptionally, one child with rotavirus diarrhea was observed to have a minimal reduction in CD4^+^ and CD8^+^ T-cell frequencies in the acute stage but had a severe reduction in CD4 and CD8 T-cell subsets at convalescence [34]. Among vaccinated children, rotavirus antigen-experienced CD4 T-cells were detected in low frequencies two weeks post-vaccination [31]. Summary findings of these studies are outlined in Table 4.

### 3.7. Proliferative, Helper and Cytotoxic T-Cells Profiles to Rotavirus Differ in Children Compared to Adults and Other Stimulants

Diminished responses and different profiles of proliferative, helper, and cytotoxic T-cell responses are elicited against rotavirus in children compared to adults or other stimulants as shown in Table 4. In a study by Jaimes et al., rotavirus-specific CD4^+^IFN-γ^+^Th1, CD4^+^IL-13^+^Th2, and CD8^+^IFN-γ^+^ cytotoxic T-cells, were investigated in children with rotavirus diarrhea in comparison to recently infected, exposed, and unexposed healthy adults. When compared, rotavirus-exposed adults had significantly higher mean proportions of rotavirus-specific Th1 and cytotoxic responses than children whose responses were similar to those observed in healthy adults. However, while the Th1 and cytotoxic T-cell responses were induced by rotavirus in both adults and children, the Th2 response was additionally observed in children with rotavirus diarrhea at a similar frequency to the Th1 response but not in adults [24]. In contrast, a study by Parra et al. showed a predominance of monofunctional CD4^+^IFN-γ^+^ and CD4^+^TNF-α^+^ Th1 response in both adults and children [30]. Another study found T-cell proliferative responses to rotavirus were generally weaker in prospectively studied children compared to adults with the adults having significantly stronger T-cell proliferation to both bovine and human rotavirus strains than any age group of children [26]. A study looking at frequencies of CD4^+^IFN-γ^+^ or IL-2^+^Th1, CD4^+^IL-13^+^Th2, CD4^+^IL-17^+^Th17 and CD8^+^IFN-γ^+^ cytotoxic T-cells in children with rotavirus and non-rotavirus diarrhea in comparison with healthy and acutely or convalescent rotavirus infected adults found similar observations. Little to no Th1, Th2, or Th17 rotavirus-specific T-cell responses were observed in children with diarrhea and few responses observed comprised Th1 and cytotoxic responses and were only observed among children with prior exposure to or existing acute rotavirus diarrhea. In contrast to children, a much larger proportion of adults, both healthy and acutely infected had detectable Th1 and cytotoxic T-cell responses [27]. These results are similar to another study that showed secretion of IFN-γ, TNF-α, GM-CSF, RANTES, MCP-1, and IL-10 from rotavirus stimulated cells in adults but not in children [30].

In comparison to other viral and bacterial stimulants, circulating rotavirus-specific T-cell responses are generally diminished. While significantly higher proliferation to rotavirus was observed in adults than children, proliferation in response to mycobacterium purified protein derivative (PPD) in children was as high as that observed in adults [26]. Among healthy children, T-cell proliferation to rotavirus was observed to be generally lower in comparison to proliferation against tetanus toxoid (TT), mycobacterium PPD antigens, and Coxsackie B4 virus (CBV) antigen [25,30]. Significantly lower frequencies of IFN-γ, TNF-α and IL-2 producing CD4 T-cells were observed against rotavirus than in response to Influenza virus antigens in children [30]

### 3.8. Rotavirus Activates Proinflammatory, Regulatory and Gut Homing Effector T-Cell Phenotypes

The T-cell immune response to rotavirus in children is characterized by an elevated activated and proinflammatory T-cell profile (Table 5). Children with rotavirus diarrhea show higher proportions of proinflammatory T-helper 17 cells complemented by higher levels of peripheral blood circulating pro-inflammatory IL-6 and IL-17 cytokines at the time of acute infection compared to healthy children [21]. Similarly, a case report of a child with rotavirus gastroenteritis reported elevated proportions of IFN-γ producing helper and cytotoxic T-cells in the acute phase of infection although these levels were reduced by convalescence [23]. Likewise, another study showed a positive correlation between T-cell proliferative responses to rotavirus and messenger ribonucleic acid (mRNA) expression of proinflammatory IFN-γ and IL-4 cytokines in healthy children [25]. Similar to these findings, a microarray analysis study of immune cell mRNA gene expression by Wang et al. revealed that children with rotavirus diarrhea had upregulation of genes encoding lymphocyte activation markers, proinflammatory cytokines, chemokines, and immune proteins in the acute stage compared to healthy children. Interestingly, although there was an elevated gene expression of lymphocyte activation markers CD69 and CD83 as well as genes encoding for the differentiation, maturation, activation, and survival of B lymphocytes, there was a reduced expression of genes involved in the proliferation, differentiation, activation, survival, and homeostasis of T lymphocytes in these rotavirus infected children [34].

The proinflammatory T-cell response to rotavirus may occur in association with either a lowered or elevated regulatory T-cell response (Table 5). Dong et al. found that rotavirus infected children had a significantly lower proportion of regulatory T-cells compared to healthy children. The lower regulatory cell profile corresponded to significantly lower levels of circulating immunosuppressive IL-10 and TGF-β cytokines [21]. In contrast, a study by Mesa et al. showed that a TGF-β dependent regulatory mechanism of rotavirus specific CD4 and CD8 IFN-γ T-cell response was absent in children with acute rotavirus gastroenteritis but present in adults, although only four and three adults were studied respectively, and showed that the lowered circulating frequency of rotavirus specific T-cells was not due to regulation by TGF-β^+^ regulatory T-cells as both rotavirus-infected and healthy children had similar proportions of these circulating Treg profiles [27]. Furthermore, another study found a positive correlation between T-cell proliferative responses and immunosuppressive IL-10 but supporting the previous studies this was not observed for TGF-β [25]. One other study also found elevated expression of other inflammation-modulating proteins IL-1R antagonist, IFN α/β receptors and IFN-stimulated proteins in rotavirus infected children [34].

Two studies reported that a substantial proportion of rotavirus-experienced T-cells express gut homing markers. As shown in Table 5, one study by Rott et al. among children convalescing after acute rotavirus infection reported higher T-cell proliferative response to rotavirus in the α4β7^hi^ lymphocyte population than α4β7^−^ lymphocyte population although this was based on cellular data obtained from one child [33]. Likewise, another study among rotavirus vaccinated children found that most of the rotavirus antigen-experienced CD4^+^ T cells expressed α4β7 gut homing marker with most cells expressing both, α4β7 and CCR9, gut homing markers [31].

## 4. Discussion

We provide an overview of the evidence and characteristics of T-cell immune responses to rotavirus in healthy, rotavirus infected, and vaccinated children. Although many research studies have been done, very few of them specifically address T-cell mediated immunity to rotavirus in children. We found only seventeen articles to include in this review.

### 4.1. Summary Findings and Implications

The majority of studies identified were within the context of rotavirus infection and only two studies assessed T-cell responses in relation to rotavirus vaccination. This is particularly surprising considering the continued development and introduction of new rotavirus vaccines [6,38] and the fact that immune correlates of protection for rotavirus vaccines remain elusive to date [7]. Additionally, the least number of studies were conducted in African children which is of concern as this region bears the highest burden of rotavirus diarrhea [3] and rotavirus vaccines within this region consistently exhibit diminished performance [17]. These findings highlight the gap in research elucidating the role of T-cell mediated immunity to rotavirus to explore their potential as immune correlates of vaccine protection and the need for a better understanding of rotavirus immune mechanisms. Such research would particularly help understand the reduced vaccine immunogenicity in African children.

T-cell immunity does play a role in the immune response to rotavirus in children. Lymphoproliferative assays provided evidence of circulating rotavirus-specific T-cells in children. The lack of proliferation observed in newborns, minimal proliferation in infants <1-year-old, and increasing proliferation with age are consistent with the exposure pattern to rotavirus in early life. However, the minimal rotavirus-specific T-cell proliferation in children aged below 1 year of age is of concern as rotavirus vaccines are administered within this period and vulnerability to rotavirus is highest in early infancy. While transplacental maternal antibody immunity is most probably important for protection in this age group, it may be necessary for new rotavirus vaccine formulations to incorporate designs allowing for enhanced T-cell activation such as the addition of adjuvants. Interestingly, evidence of rotavirus T-cell proliferation is also seen in some newborns that could be a result of in-utero or very early exposure to rotavirus antigens and is of significance for neonatal rotavirus vaccines strategies. Rotavirus vaccines administered at birth have been developed and found to be safe and highly efficacious in newborns. This birth dose vaccination could potentially impart rotavirus-specific memory T-cells thus providing an opportunity for cell-mediated protection very early on in life [39]. This early protection would have a considerable impact on further reduction of rotavirus burden in low-income countries where a sizeable proportion of children are infected with rotavirus before receipt of the first vaccine dose that has been associated with poor vaccine seroconversion [11,40].

Broadening of cross-reactive T-cells with increasing age is consistent with exposure to different rotavirus strains as children age. These results further implied that rotavirus-specific T-cells recognize epitopes shared by different infecting rotavirus serotypes indicating that T-cell immunity can provide cross-reactive protection. Rotavirus has a large strain diversity based on varying combinations of G- and P-serotypes and genotypes classified by antibody reactivity to VP7 and VP4 viral proteins respectively [8]. Rotavirus strains that cause infections in humans and commonly infect children aged <5 years are well known but evolutionary genetic mutation and reassortments eventually give rise to new strains [41]. This observed T-cell proliferation irrespective of infecting G-serotype suggests that rotavirus induced T-cell immunity in children is not G-serotype specific which is important for effective vaccine strategies. For instance, Rotarix, a monovalent G1P [8] rotavirus vaccine has shown protection against non-vaccine serotype rotavirus strains, however, vaccine strain breakthrough still occurs and the extent to which this cross-reactive immunity is mediated by T-cells or antibody responses is unclear and needs further investigation [42]. Total circulating antibody and homotypic and heterotypic neutralizing antibodies are associated but not entirely correlated with protection, which has suggested that other immune mechanisms like these cross-reactive T-cells are likely at play [7].

The available literature shows that both memory B and T-cell immunity are developed after rotavirus exposure with T-cell responses occurring in tight association with the antibody response. This review revealed more frequently detected T-cell responses in children that were seropositive than those seronegative for rotavirus-specific antibodies as well as in secondary versus primary infections. However, the antibody response is more persistent and due to the more transient nature of the T-cell response, T-cell immunity detected in children most likely reflects previous rather than active exposure. Therefore, in infants, T-cell immunity may be more useful as a measure of child-specific immune memory and in early infancy to discriminate from passively acquired maternal immune memory in response to infection. Additionally, in the context of vaccination, detection within shorter time periods post-vaccination would be required in the assessment of these effector T-cell responses. Nevertheless, the detection of both T-cells and antibody responses is necessary to adequately describe the immune response to rotavirus in children infection.

Evidence of T-cell proliferation in the absence of increasing antibody titers in some children speaks towards the existence of anti-rotavirus protection mediated via a direct T-cell immune effector in children. The direct effector contribution of T-cells has been shown in murine model depletion and adoptive studies where depletion of CD8 T-cells resulted in the delayed rate of resolution of rotavirus infection, CD4 T-cell depletion was associated with chronic viral shedding and complete loss of protection [14], and adoptive transfer of rotavirus primed CD4 and CD8 T-cells resulted in shorter rotavirus shedding [43]. In such murine studies, a significant loss of protection against rotavirus has also been observed in T-cell deficient and T-cell receptor (TCR) knockout mice with the delayed resolution of rotavirus infection attributed to the depletion of the CD4^+^ T-cell subset, while B-cell and TCR deficient mice remained protected [15]. In this review, direct effects of T-cell immunity were exemplified by the impaired rotavirus antibody response, chronic viral shedding, and inability to clear infection observed in T-cell immunodeficient children. In the context of vaccination, it is plausible that lowered antibody responses detected in non-seroconverting children based on fold change in antibody response may not entirely imply reduced protection as T-cell immunity may provide direct protective and immune memory functions. The contribution of T-cell immune memory in the measurement of vaccine immunogenicity may have implications for measures of vaccine efficacy.

The positive association between higher rotavirus CD4 T-helper cell response and rotavirus seropositivity or higher neutralizing IgG in children highlights the particular importance of indirect protection offered via the CD4 T-cell helper function in the production of the antibody response. In adoptive transfer murine models, rotavirus primed CD4 T-cells and not CD8 T-cells are associated with increased production and maintenance of secretory IgA that is important in mucosal immunity, and both serum IgA and IgG are currently recognized as valuable surrogate endpoints for protection [12]. Therefore, taking this into account, in regions of poor rotavirus vaccine performance, there is a need for elucidating detailed profiles of these CD4 T-cells in relation to the magnitude and neutralizing ability of the antibody responses among vaccinated children. Magnitude and maintenance of antibody response may be reliant on characteristics of the elicited CD4 T-cell response. Such T-cell studies may provide useful insights for the observed lower vaccine immunogenicity and effectiveness trends in these regions.

In children, these characteristics of CD4 and CD8 T-cell responses to rotavirus include predominantly Th1 but also Th17 responses. Activated CD4 and CD8 T-cells secreting proinflammatory cytokines particularly IFN-γ and IL-17 appear important in this immune response. IFN-γ cytokine has direct anti-viral effects and IL-17 is associated with the provision of protection via recruitment of other immune cells with both cytokines shown to be important in the clearance of rotavirus infection [44]. On the other hand, regulatory T-cells which may suppress the proinflammatory immune response in efforts to maintain homeostasis also occur in response to rotavirus. The regulatory T-cells can have a negative or positive influence on the immune response to rotavirus infection or vaccination. This review revealed IL10^+^ and FOXP3^+^ regulatory T-cells as distinct subpopulations with opposing effects on rotavirus antibody immunity. In this context, a distinct population of CD4^+^/CD8a^+^ CCR6^+^CXCR6^+^ Treg cells has been identified in the human colon, which responds to fecal bacterial species and produces IL-10 [45]. These cells could indeed drive distinct outcomes during rotavirus infection compared to their FOXP3^+^ Treg counterparts. For live attenuated rotavirus vaccines, assessing these Th1 and Th17 inflammatory and FOXP3^+^ and IL-10^+^ regulatory T-cell profiles in children may provide insights into the observed vaccine immunogenicity.

In addition to these conventionally studied CD4 and CD8 T-cell subsets, recently identified innate-like T-cells such as the gamma delta T-cell (γδT), mucosal-associated invariant T-cells (MAIT), and natural killer T-cells (NKT) are enriched in mucosal tissues and have been reported to provide protective effector activities against human intestinal infections. Through direct cytokine action or indirectly via recruitment of other immune effector cells cytokine responses, these innate-like T-cells have been suggested to provide early antiviral immune protection in the interface between innate immunity and induction of adaptive immunity and have been associated with inhibited viral replication of important human viral pathogens [46,47]. There is an urgent need to also consider the characterization of these atypical T-cell profiles and how they relate to conventional CD4 and CD8 T-cell subsets in relation to observed rotavirus infection or vaccine immunogenicity.

Circulating rotavirus-specific T-cells in children are generally low in frequency during the acute than convalescent phase and much weaker than those generated in adults and against other pathogens. The lowered frequency of rotavirus-specific T-cells in the initial response may be a direct consequence of their migration from circulation to gut mucosal priming sites to carry out effector function. This is supported by literature documenting higher T-cell proliferation within α4β7^hi^ subset and a higher proportion of CD4 T-cells responding to rotavirus expressing α4β7 or CCR9 gut homing markers. Current live attenuated oral rotavirus vaccines aim to mimic natural infection immune priming within the gut. The extent to which such vaccines elicit these gut homing effector T-cell phenotypes may relate to the protective effect of vaccination. With new parenterally administered rotavirus vaccines being introduced, their ability to elicit these gut homing phenotypes must also be studied. While murine models have documented the development of mucosal immunity from parenteral vaccination [48], the generation of gut homing rotavirus specific T-cells in children vaccinated with parenteral rotavirus vaccine remains to be determined although an observed reduction in viral shedding in clinical trials conducted thus far has implied generation of local mucosal effectors [49]. It will, therefore, be important to conduct studies assessing the homing phenotypes elicited by rotavirus vaccination which may influence effector abilities in the protection against rotavirus at the gut.

When compared to tuberculin, tetanus toxoid, and influenza-derived antigens for which childhood vaccines are also administered, the T-cell responses induced by rotavirus antigen were observed to be diminished. Reasons for such variations in antigen-specific responses in early life can include immune dysfunction in antigen-specific presentation and differences in antigen-specific T-cell activation, proliferation, and effector versus memory generating functions. A better understanding of these T-cell phenotypes responding to rotavirus in this context has the potential to be exploited for improved immunity [50]. Considering the role of T-cell phenotypes in the child’s immune response to infection or vaccinations, it should be important when assessing immune responses in children to account for pathogens that have a strong modulatory effect on these T-cell populations. For instance, cytomegalovirus, a ubiquitous pathogen, and potent T-cell modulator have been shown to influence immune and vaccine-induced T-cell profiles in children [51,52] but data is unavailable on its modulatory effect on anti-rotavirus T-cell immunity in children.

### 4.2. Strengths and Limitations

To the best of our knowledge, this is the first systematic review of the T-cell response to rotavirus in children using a clearly defined search and screening strategy to obtain existing literature. Our review gives an overview of research done prior to and post introduction of rotavirus vaccines and provides evidence supporting the need for more research on T-cell mediated immunity in children not only as it relates to infection but also vaccination. This review provides current knowledge in the literature on different subsets and characteristics of T-cells response to rotavirus encompassing general proliferation, specific phenotypes, functional cytokine secretion, and migratory profiles. The review also covered the relationship of T-cell responses to widely studied antibody responses.

Limitations in this review primarily arose from the nature of the studies identified. A substantial proportion of studies, particularly those conducted earlier, reported lymphoproliferative activity as an indication of T-cell immunity. However, caution must be taken in their interpretation as the detected proliferation potentially includes that of innate and B-cells. Lymphoproliferative-based measures, while giving insights to T-cell immunity, do not provide specific T-cell immune data in comparison to current more advanced techniques such as multicolor flow cytometry. Additionally, aside from four studies, the majority were conducted within the last decade and as such did not utilize more recent immunological methods such as higher cell marker parameter flow cytometry to provide more comprehensive T-cell knowledge.

Another limitation is that the studies identified used a diverse range of immune stimulants to assess the rotavirus T-cell responses which included different rotavirus strains or mitogens and had variations in reporting format for the T-cell outputs. This introduced large methodological heterogeneity that presented a major challenge in the quantitative synthesis of the evidence that was provided. Additionally, there was a lack of sufficient reporting of statistical data in several studies and more so in studies conducted much earlier on, and for some studies, sample sizes were very small making generalization of findings difficult.

## 5. Conclusions

T-cells clearly have a role to play in the immune response to rotavirus in children. This review shows that these responses are heterotypic and although present at low circulating levels and less persistent than antibodies, can be detected in children and develop through repeated exposure. Both CD8 and CD4 T-cell subsets are involved in this response and are primarily of a Th1 and gut homing phenotype. However, there is a paucity of T-cells studies, wide methodological differences, and a lack of sufficient quantitative data sets directly associating T-cell immunity to protection from rotavirus infection or in relation to immunogenicity of rotavirus vaccines. Thus, it is imperative that further research be done investigating T-cell responses against rotavirus and the standardization of rotavirus-specific T-cells assays is needed in this population.

Africa bears a disproportionate burden of rotavirus diarrheal disease and has an urgent need for research in this area. Such studies may also establish whether the observed lower vaccine-induced anti-rotavirus antibodies in African children could be attributed to limited or impaired T-cell responses. There is also a need to address innate-like T-cell subsets and the inclusion of more phenotypic markers using more developed immunological assays to provide comprehensive T-cell immunology data. In rotavirus vaccinology, it will be important to assess T-cell immunity relationship to seroconversion rates and clinical protection against rotavirus infection. Such research could form a good basis for further exploration of T-cells as a potential immune correlate of protection and inform the development of next-generation vaccines.

## Figures and Tables

**Table 1:** Characteristics of studies included in the systematic review.

Author Year [Ref]	Country	Design	Child Population, *n*	Age	Rotavirus Exposure	T-Cell Stimulant	T-Cell Detection Method	T-Cell Response Markers Evaluated
Dong et al., 2015 [21]	China	Observational	RV-AGE, *n* = 102; Healthy, *n* = 30	3 mos to 3 yrs; 2 mos to 3 yrs	Rotavirus infection	PMA Ionomycin	Flow Cytometry	Treg (CD4^+^CD25^+^) Th17 (CD4^+^IL-17^+^)
Elaraby et al., 1992 [22]	Egypt	Observational	RV-AGE, *n* = 6; Healthy, *n* = 50	NR; Newborn, 1 to <12 mos, 12 to 24 mos, 24 to 60 mos	Rotavirus infection	Rotavirus antigen, PHA	Light microscopy, Indirect Fluorescent Microscopy	Stimulation Index, positive > 1.5 Helper/suppressor (CD4:CD8) ratio CD3, CD4, CD8 subsets
Iwasa et al., 2008 [23]	Japan	Observational	RV-AGE, *n* = 1	6 mos	Rotavirus infection	Nil	Flow Cytometry	CD4^+^/CD8^+^IFN-γ^+^ CD4^+^/CD8^+^ CD4^+^/CD8^+^ ratio
Jaimes et al., 2002 [24]	Colombia	Observational	RV-AGE, *n* = 12	6 mos to 7 yrs	Rotavirus infection	RRV, SEB, CD28, CD49d	Flow Cytometry	CD4/CD8 CD69^+^IFN-γ^+^, CD4/CD8 CD69^+^IL-13^+^
Makela et al., 2006 [25]	Finland	Observational	Healthy (T1D at risk), *n* = 183	≤15 yrs	N/A	HRV Wa, BRV NCD, CBV, PPD, TT, PHA, PCB	Proliferation assay, PCR	Stimulation Index, positive ≥ 3 IFN-γ^+^, IL-4^+^, IL-10^+^ and TGF-β^+^ PBMC gene expression
Makela et al., 2004 [26]	Finland	Observational	Healthy children (T1D at risk), *n* = 20	3 mos to 5 yrs	N/A	HRV Wa, BRV NCD, PPD, TT, PHA	Proliferation assay	Stimulation Index, positive ≥ 3
Mesa et al., 2010 [27]	Colombia	Observational	RV-AGE, *n* = 17; Non-RV-AGE, *n* = 36	Median 14 mos (range 4 to 22 mos)	Rotavirus infection	HRV Wa, Simian RRV, SEB, CD28, CD49d	Flow Cytometry	CD4^+^ and CD8^+^ CD4^+^/CD8^+^IL-2^+^, IL-10^+^, IL-13^+^, IL-17^+^, IFN-γ^+^ CD4^+^CD25^+^,CD4^+^CD25^+^CD127^low^, CD4^+^CD25^+^CD127^low^TGF-β^+^
Offit et al., 1992 [28]	USA	Observational	Healthy, *n* = 48	Newborn to 18 yrs old	N/A	HRV Wa, HRV HCR3a strains, Simian RRV, concanavalin A	Proliferation assay	Stimulation Index, positive ≥ 3:1
Offit et al., 1993 [29]	USA	Observational	RV-AGE, *n* = 8	<2 yrs old.	Rotavirus infection	HRV HCR3a, HRV W179	Proliferation assay	Stimulation Index, positive ≥ 3:1
Parra et al., 2014 [30]	Colombia	Observational	Healthy, *n* = 5	2 to 8 yrs old	N/A	Simian RRV, Influenza vaccine, TT, SEB, CD28, CD49d	Flow Cytometry, Proliferation assay	CD4^+^/CD8^+^IL-2^+^IFN-γ^+^, TNF-α^+^ IFN-γ, TNF-α, GM-CSF, RANTES MCP-1 and IL-10, IL-4, IL-6, IL-17A, IL-9, and IL-2 secreting PBMC CD4^+^ and CD8^+^ proliferation
Parra et al., 2014 [31]	Colombia	Randomised Controlled Trial	Vaccine, *n* = 35	2 to 4 mos	Rotavirus vaccination	RRV, NSP2, VP3-4, VP6-7, SEB, CD28, CD49d	Flow Cytometry	VP6-7 tetramer^+^ CD62L^−^CD45RA^+^/^−^ and CD62L^+^CD45RA^−^CD4^+^, Gut homing (α4β7^+^ and CCR9^+^) VP6-7 tetramer^+^ CD4^+^
			Placebo, *n* = 24					
Rojas et al., 2003 [32]	Colombia	Observational	RV-AGE, n= 15; Non-RV-AGE, *n* = 13	3 mos to 7 yrs	Rotavirus infection	RRV, SEB, CD28, CD49d	ELISpot	IFN-γ^+^, IL-4^+^ secreting PBMC, IFN-γ^+^, IL-4^+^ secreting CD4^+^ and CD8
Rott et al., 1997 [33]	USA	Observational	RV-AGE, *n* = 1	NR	Rotavirus infection	RRV, concanavalin A	Flow Cytometry, Proliferation assay	β7^+^ and β7^−^ PBMC Stimulation Index
Wang et al., 2007 [34]	USA	Observational	RV-AGE, *n* = 10; Healthy, n= 8	<3 yrs	Rotavirus infection	Nil	Flow Cytometry PCR	CD4^+^/αβCD4^+^, and CD8^+^/αβCD8^+^, CD4^+^/CD8^+^CD69^+^, and CD4^+^/CD8CD83^+^ CD1C, CD2, CD3D, CD28, CD96, CD2, αβ TCR, Lck and Lck substrate, LAT, SLP-76, IL-16, CD27, IL-17R, IL-27Rα, IL-7R, RP1, LIGHT, and MAL gene expression
Weinberg et al., 2018 [35]	Botswana, Tanzania, Zambia, Zimbabwe	Randomised Controlled Trial	Vaccine, *n* = 42; Placebo *n* = 47	2 to ≤15 wks	Rotavirus vaccination	Nil	Flow Cytometry	CD4^+^ CD4^+^IL-10^+^ CD4^+^FOXP3^+^CD25^+^CD8^+^FOXP3^+^CD25^+^
Wood et al., 1988 [36]	England	Observational	RV-AGE, *n* = 2	Newborn and 11 mos	Rotavirus infection	Simian RRV SA11strain, PHA	Proliferation assay	Stimulation Index, positive > 2, T-cell frequency
Yasukawa et al., 1990 [37]	Japan	Observational	Healthy child, *n* = 1	Newborn	N/A	HRV Wa strain, BRV NCD strain	Proliferation assay	Scintillation count/minute

Abbreviations: α4β7 = alpha 4 beta 7. β7 = beta 7. BRV = bovine rotavirus. CBV = Coxsackie B4 virus. CCR9 = C-C motif chemokine receptor 9. CD1C = cluster of differentiation 1C. CD2 = cluster of differentiation 2. CD3 = cluster of differentiation 3. CD3D = cluster of differentiation 3D. CD4 = cluster of differentiation 4. αβCD4 = alpha beta cluster of differentiation 4. CD8 = cluster of differentiation 8. αβCD8 = alpha beta cluster of differentiation 8. CD25 = cluster of differentiation 25. CD27 = cluster of differentiation 27. CD28 = cluster of differentiation 28. CD45RA = cluster of differentiation 45RA. CD49d = cluster of differentiation 49d. CD62L = cluster of differentiation 62L. CD69 = cluster of differentiation 69. CD83 = cluster of differentiation 83. CD96 = cluster of differentiation 96. CD127 = cluster of differentiation 127. CD28 and CD49d were used as co-stimulators. ELISpot = enzyme linked Immunospot. FOXP3 = Forkhead box protein P3. GM-CSF = granulocyte-macrophage colony-stimulating factor. HCR3a = human cytopathic rotavirus 3a. HRV = human rotavirus. IFN-γ = interferon gamma. IL2 = interleukin 2. IL4 = interleukin 4. IL6 = interleukin 6. IL7R = interleukin 7 receptor. IL9 = interleukin 9. IL10 = interleukin 10. IL13 = interleukin 13. IL16 = interleukin 16. IL17 = interleukin 17. IL17R = interleukin 17 receptor. IL27R α = interleukin 27 receptor alpha subunit. LAT = linker for activation of T cells. Lck = lymphocyte-specific protein tyrosine kinase. LIGHT = tumor necrosis factor superfamily member 14. MAL = myelin and lymphocyte protein. MCP1 = monocyte chemoattractant protein 1. mos = months. N/A = not applicable. NCD = Nebraska Calf Diarrhoea. NR = not reported. NSP2 = non-structural protein 2. PBMC = peripheral blood mononuclear cells. PCB = purified Coxsackie B4 virus. PCR = polymerase chain reaction. PHA = phytohemagglutinin. NR = not reported. PMA = phorbol myristate acetate. PPD = tuberculin purified protein derivative. RANTES = regulated on activation, normal T-cell expressed and secreted. RP1 (synonym MAPRE2) = microtubule associated protein RP/EB family member 2. RRV = rhesus rotavirus. RV-AGE = rotavirus acute gastroenteritis. SEB = staphylococcal enterotoxin B. SLP-76 = Src homology 2 domain-containing leukocyte protein of 76 kilodalton. TCR = T-cell receptor. αβTCR = alpha beta T-cell receptor. T1D = type 1 diabetes. TGF-β = transforming growth factor beta. Th17 = T helper 17. TNF-α = tumor necrosis factor alpha. Treg = regulatory T-cell. TT = Tetanus Toxoid. USA = United States of America. yrs = years. wks = weeks. VP6-7 = viral protein 6-7.

**Table 2:** Relationship between rotavirus T-cell proliferation and child age.

Author, Year [Ref]	Child Age	T-Cell Response	Key Findings	Interpretation
Elaraby et al., 1992 [22]	RV-AGE: (*n* = 6), age NR Healthy: birth (*n* = 14), 1 to <12 mos (*n* = 14), 12 to <24 mos (*n* = 10), and 24 to <60 mos (*n* = 12).	Lymphoproliferation against rotavirus antigen (strain NR)	Lymphoproliferation in all 6/6 (100%) children with RV-AGE versus 18/50 (36%) in healthy children. No lymphoproliferation in newborns but increasing lymphoproliferation in older age groups from 2/14 (14%) in 1 to <12 mos, to 5/10 (50%) in 12 to <24 mos and up to 11/12 (92%) in 24 to <60 mos age groups. Mean (SD) lymphoproliferation lowest at birth 1.11 (0.16) and in the 1 to <12 mos age group 1.08 (0.22), increased to 1.5 (0.72) in the 12 to <24 mos age group and highest in the 24 to <60 mos age group at 3.58 (1.66)	Rotavirus is an effective T-cell inducer T-cell immunity to rotavirus increases with age.
Makela et al., 2004 [26]	Healthy: (*n* = 20), 3 months to 60 months age: sampled 3 mos to 6 mos (*n* = 23), 9 mos to 12 mos (*n* = 26), 15 mos to 24 mos (*n* = 65), 27 mos to 36 mos (*n* = 38), 39 mos to 48 mos (*n* = 31), 51 mos to 60 mos (*n* = 11)	Lymphoproliferation against bovine NCD (P serotype 6, G serotype 6) and human purified and lysate Wa (P serotype 1, G serotype 1A) rotavirus strain	Lymphoproliferation against both human and bovine rotavirus antigens are more common with increasing age (NS, Fisher’s exact test) Positive correlation between lymphoproliferation against bovine and human lysate rotavirus (*p* < 0.0001, r_s_ = 0.52, Spearman correlation test) and between bovine and purified human rotavirus *p* < 0.0001, r_s_ = 0.56, Spearman correlation test)	T-cell immunity to rotavirus increases with age and is cross-reactive
Makela et al., 2006 [25]	Healthy: (*n* = 183), age range 3.5 yrs to 11.3 yrs	lymphoproliferation against human Wa (P serotype 1, G serotype 1A) and bovine NCD (P serotype 6, G serotype 6) rotavirus strains	Lymphoproliferation positively correlated with age for human (*r* = 0.32, *p* < 0.0001) and bovine (*r* = 0.20, *p =* 0.001) rotavirus	T-cell immunity to rotavirus increases with age and is cross-reactive
Offit et al., 1992 [28]	Healthy: age groups newborns (*n* = 11), 16 days to <6 mos (*n* = 11), 6 mos to <2 yrs (*n* = 8), 2 yrs to 5 yrs (*n* = 8), 5 yrs to 18 yrs (*n* = 10).	lymphoproliferation against human Wa (serotype 1) and HCR3a (serotype 3) and simian rhesus rotavirus strain 2 (serotype 3) antigens	Few 1/11 (9%) children aged <6 mos had lymphoproliferation against human rotavirus but unexpectedly 4/11 (36%) newborns showed lymphoproliferation against both human and simian rotavirus antigens In contrast, 6/8 (75%) and 4/8 (50%) children aged between 6 mos to 2 yrs and 10/13 (77%) and 6/16 (38%) aged between 6 mos to 5 yrs had lymphoproliferation against human rotavirus and simian rotavirus antigens respectively. In children aged >5 yrs old, ~80% had lymphoproliferation against both human and simian rotavirus antigens	T-cell immunity to rotavirus increases with age and is cross-reactive. T-cell immunity to rotavirus may occur at birth due to maternal transfer or in-utero rotavirus exposure
Offit et al., 1993 [29]	RV-AGE: *n* = 8, <2 yrs caused by serotype 1 (P type 1, G type 1, *n* = 2), serotype 3 (P type 1, G type 3, *n* = 3) and serotype 4 (P type 1, G type 4, *n* = 3) rotavirus strains and followed up in convalescence and late convalescence	Lymphoproliferation against human WI79 (P-type 1, G type 1) and HCR3a (P-type non-human, G type 3) rotavirus strain antigens	During the acute stage, a few 1/8 (13%) children had lymphoproliferation against WI79 rotavirus antigen. In contrast during convalescence most 6/8 (75%) *p* < 0.05 and 5/6 (83%) *p* < 0.05 children had lymphoproliferation against WI79 and HCR3a rotavirus strains respectively; 4/6 (67%) had lymphoproliferation to both strains, 1/6 (17%) to only HCR3a, 2 to only WI79, while no reactivity to either strain was observed in 1/6 (17%) children studied. At late convalescence, all 4/4 (100%) children studied had lymphoproliferation. No proliferative response specific for the G type of the infecting rotavirus strain in either convalescent or late convalescent children was observed	T-cell immunity is present during acute and convalescent rotavirus infection. T-cell immunity to rotavirus is not G-type specific and may recognize T-cell epitopes shared by different rotavirus strains
Yasukawa et a., 1990 [37]	Healthy full-term newborn	Lymphoproliferation against human Wa (serotype 1) strain rotavirus antigen	Lymphoproliferation against human rotavirus antigen absent in the newborn	T-cell immunity to rotavirus occurs in an antigen-specific manner

Abbreviations: HCR3a = human cytopathic rotavirus 3a. mos. = months. NCD = Nebraska Calf Diarrhoea. NR = not reported. NS = not statistically significant. *p* = probability value. RV-AGE = rotavirus acute gastroenteritis. *r* = Pearson’s correlation coefficient. r_s_ = Spearman’s rank correlation coefficient. SD = standard deviation. yrs = years.

**Table 3:** Rotavirus T-cell proliferation, frequencies, and phenotypes in relation to an antibody response.

Author, Year [Ref]	Population and Antibody Response	T-Cell Response	Key Findings	Interpretation
Makela et al., 2006 [25]	Healthy: rotavirus IgA and/or IgG seropositive (*n* = 112) or rotavirus seronegative (*n* = 41)	Lymphoproliferation and IFN-γ producing PBMC against purified and lysate human and bovine rotavirus antigens	Seropositive children had more frequent lymphoproliferation 50/112 (45%) than seronegative 4/41, 10% children *(p* < 0.0001) and stronger lymphoproliferation against purified (*p* = 0.010), lysate (*p* = 0.0031) human rotavirus and bovine rotavirus (*p* < 0.0001) Seropositive children had higher IFN-γ producing PBMC compared to seronegative children (*p* = 0.084)	Prior exposure to rotavirus induces both memory T-cell and B-cell immunity in children.
Makela et al., 2004 [26]	Healthy: rotavirus IgA and/or IgG seropositive or seronegative at 3 mos to 12 mos of age with primary (*n* = 19) or secondary (*n* = 5) rotavirus infections	Lymphoproliferation against purified and lysate human rotavirus	Minimal or absent proliferation in children with low rotavirus antibody titers. Increase in antibody titers accompanied by stronger lymphoproliferation against lysate and purified human rotavirus (*p* = 0.017 and *p* = 0.027, respectively, Wilcoxon test) and more positive lymphoproliferation in 9/24 (37.5%) cases. In contrast, one child had lymphoproliferation without a simultaneous increase in rotavirus antibody titers. Lymphoproliferation more frequent in secondary infections than primary infections (NS) Rotavirus-specific antibody levels remained elevated throughout follow-up after rotavirus infection but lymphoproliferation declined shortly after infection and was detectable less than 12 months after primary infection (mean 5 months)	T-cell immunity occurs in tight association with rotavirus antibody response. T-cell immunity can occur in absence of detectable increasing antibody response More persistent and stronger T-cell immunity develops after repeated rotavirus exposure Unlike antibodies, T-cell immunity to rotavirus is transient.
Offit et al., 1992 [28]	Healthy: age groups newborns (*n* = 11), 16 days to <6 mos (*n* = 11), 6 mos to <2 yrs (*n* = 8), 2 yrs to 5 yrs (*n* = 8), and 5 yrs to 18 yrs (*n* = 10) with rotavirus neutralising antibody	Lymphoproliferation against human and simian rotavirus	More newborns and children < 6 mos, had neutralizing antibodies against at least one rotavirus strain than lymphoproliferation In contrast, among older age groups between 6 mos and 18 yrs, most children had both lymphoproliferation and rotavirus neutralizing antibodies to at least one human or simian rotavirus strain.	Development of T-cell immunity to rotavirus occurs in conjunction with the development of antibody responses in children In young infants aged <6 mos measurement of T-cell immunity is possibly more reliable in discriminating active from a passively acquired immune response Both T-cell and antibody immunity induced by rotavirus in children can be cross-reactive
Offit et al., 1993 [29]	RV-AGE: caused by P-type 1 and different G type strains followed up in convalescence and late convalescence with rotavirus IgA and neutralizing antibodies (*n* = 8)	lymphoproliferation against human rotavirus	Neutralizing antibodies were mounted against different P and G serotype infecting rotavirus strains and similarly, lymphoproliferation was also mounted against different infecting G serotypes strains	Both rotavirus specific neutralizing antibody and T-cell immunity in children may not clearly distinguish P and G infecting serotypes
Parra et al., 2014 [31]	Rotavirus IgA seropositive vaccinated (*n* = 35) and seronegative placebo (*n* = 24)	Frequency of CD4 T-cells positive for rotavirus specific VP6-7 T-cell epitope	Vaccinated seropositive children had a higher frequency of VP6-7 tetramer-positive activated CD4 T-cells (40–71%) than placebo seronegative children (0–8%)	Rotavirus-specific antibody responses to vaccination are accompanied by rotavirus-specific CD4 T-cells in children.
Weinburg et al., 2018 [35]	PHEU and PHIV (*n* = 42) vaccinated with pentavalent live rotavirus vaccine: IgA and neutralizing IgG	Frequency of several CD4 and CD8 T-cell phenotypes	Higher CD4 T-cell frequency and counts marginally and significantly associated with higher IgG neutralizing antibodies to 3/5 viral strains tested Higher frequencies of CD4^+^FOXP3^+^CD25^+^ and CD8^+^FOXP3^+^CD25^+^ regulatory T-cells were marginally or significantly (*p* < 0.1) associated with higher rotavirus IgG neutralizing antibodies to 4/5 viral strains in the RV5 vaccinated and significantly associated with higher IgA antibodies. These associations remained at least marginally significant after adjustment for CD4 T-cell proportions. Significant negative correlations with antibody titers were observed for CD4^+^IL10^+^ regulatory T-cells	Rotavirus CD4 T-cells are induced in positive association with the antibody response to vaccination FOXP3^+^CD25^+^ regulatory CD4 and CD8 T-cells may positively influence antibody responses by the protection of B cells against intense activation and apoptosis while IL10^+^ regulatory CD4 T-cells may negatively influence this response by downregulation of immune responses via bystander mechanisms.
Wood et al., 1988 [36]	CHH (*n* = 1) and CHARGE associated (*n* = 1) T-cell deficiency and rotavirus IgG	Lymphoproliferation against mitogens, rotavirus antigen, and proportions of T-cells	Poor lymphoproliferation and absent rotavirus specific IgG antibody response associated with persistent rotavirus diarrhea.	Rotavirus-specific T-cell deficiency is associated with impaired antibody response and inability to clear rotavirus infection

Abbreviation: CD4 = cluster of differentiation 4. CD8 = cluster of differentiation 8. CD25 = cluster of differentiation 25. CHH = cartilage hair hypoplasia. CHARGE = coloboma, heart defects, atresia choanae growth retardation, genital abnormalities, and ear abnormalities. FOXP3 = Forkhead box protein P3. IFN-γ = Interferon gamma. IgA = Immunoglobulin A. IgG = Immunoglobulin G. IL1–10 = Interleukin 10. mos. = months. NS = not significant. PBMC = peripheral blood mononuclear cells. PHEU = perinatally HIV exposed but uninfected. PHIV = perinatally HIV infected. *p* = probability value. RV-AGE = rotavirus acute gastroenteritis. RV5 = pentavalent rotavirus vaccine. VP6-7 = viral protein 6-7. yrs = years.

**Table 4:** Proliferative, Helper, and cytotoxic T-cell frequency to rotavirus in children compared to adults and other stimulants.

Author, Year [Ref]	Population	T-Cell Response	Key Findings	Interpretation
Elaraby et al., 1992 [22]	Healthy: (*n* = 50); RV-AGE: (*n* = 6)	CD3 (OKT3 pan), CD4 (OKT4 helper), CD8 (OKT8 frequency, CD4:CD8 T-cell ratio	Depressed CD4 T-cell frequency (33.4%) and a lower CD4:CD8 ratio (1.36) in children with rotavirus diarrhea compared to normal CD4 (range 47.1% to 55.7%) and CD8 (23.8% to 25%) T-cell frequency and helper: suppressor ratio (1.9 to 2.23) in healthy children	Lowered CD4 T-cells during acute infection may be a result of CD4 T-cell migration out of circulation to effector sites
Iwasa et al., 2008 [23]	RV-AGE: (*n* = 1)	CD4 and CD8 T-cell frequency, CD4:CD8 T-cell ratio	Depressed CD4 T-cells frequency (15.7%) and lowered CD4:CD8 ratio (0.41) but normal CD8 T-cell frequency (38.76%) in acute phase. Depressed CD4 T-cells frequency (14.55%) and lowered CD4:CD8 ratio (0.42) sustained in early convalescence but normalized in late convalescence	Lowered CD4 T-cell during acute infection may be a result of CD4 T-cell migration out of circulation to effector sites CD4 T-cells may be more critical effectors than CD8 T-cells in mucosal tissue sites
Mesa et al., 2010 [27]	Non-RV-AGE seronegative (*n* = 15) or seropositive (*n* = 21) and RV-AGE (*n* = 17) children. Healthy (*n* = 21) and RV-AGE adults (*n* = 5)	Lymphopenia and Th1, Th2, Th17 CD4 and cytotoxic CD8 T-cells	Absolute lymphopenia in 5/12 (41.6%) children with RV-AGE compared to only 1/25 (4%) in children with non-RV-AGE Low (<0.06) or undetectable frequencies of IFN-γ^+^, IL-13^+^, IL-2^+^, IL-10^+^ and IL-17^+^CD4 T-cells in most children with non- and RV-AGE. The IFN-γ^+^CD4 and CD8 T-cells were observed in a few 2/12 children with previous rotavirus exposure or rotavirus diarroea. In contrast, higher frequencies (≤0.65%) of rotavirus-specific CD4^+^IFN-γ^+^ and CD4^+^IL-2^+^ T cells were detected in the majority 14/21 (66.7%) and 6/10 (60%) of healthy adults, respectively. Similarly, CD8^+^IFN-γ^+^ and CD8^+^IL-2^+^ T cells were observed in 8/20 (40%) and 1/9 healthy adults, respectively.	Low circulating frequency of Th1, Th2, Th17, and cytotoxic T-cells in acute rotavirus that may result from effector T-cell functions at mucosal sites of infection Diminished rotavirus Th1 and cytotoxic responses in children compared to adults
Parra et al., 2014 [31]	Seropositive vaccinated: (*n* = 35) and seronegative placebo: (*n* = 24)	Rotavirus (VP6-7 tetramer) antigen experienced CD4 T-cells	Low frequency (0.001–0.1%) rotavirus antigen experienced CD4 T cells in children two weeks post two-dose vaccination	CD4 T-cells are expanded after rotavirus vaccination but low circulating frequency
Wang et al., 2007 [34]	RV-AGE: (*n* = 10); Healthy (*n* = 8)	Lymphopenia, frequencies of CD4, αβ^+^CD4, CD8 and αβ^+^CD8 T-cells	Lymphopenia in majority 5/7 (71%) of children with RV-AGE and repressed T-cell proliferation, differentiation, activation, survival, and homeostasis mRNA gene expression Lower mean frequency of CD4 (20%, range 10.4% to 26.8%) and αβ^+^CD4 (17% range 9% to 22.6%) T-cells in RV-AGE than in healthy children (50.9% range 38.6% to 60.5%) and (46.8% range. 36.7% to 53.7%) respectively (*p* < 0.01). CD4 T-cell frequencies significantly increased (*p* < 0.01) to that of healthy children at convalescence. Similarly, lower mean frequency of CD8 (2.8%, range 1.6% to 3.8%) and αβ^+^CD8 (2.9%, range 1.7% to 3.7%) T-cells in RV-AGE than in the healthy children (10.9%, range 7.4% to 13.5%) and (8.6%, range 6.1% to 10.5%) respectively (*p* < 0.05). Both CD8 and αβ^+^CD8 T-cell frequencies significantly increased (*p* < 0.01) to that of healthy children at convalescence.	Altered T-cell homeostasis and low circulating frequency of CD4 and CD8 T-cells in acute rotavirus that may result from effector T-cell functions at mucosal sites of infection
Jaimes et al., 2002 [24]	RV-AGE children (*n* = 12), rotavirus exposed asympomatic and symptomatic adults (*n* = 19), healthy adults (*n* = 7)	Th1 and Th2 CD4 and cytotoxic CD8 T-cell frequencies	Lower mean rotavirus specific CD8 IFN-γ T-cell frequency 0.02% (SEM 0.007% range −0.01 to 0.08%) in RV-AGE children than exposed adult 0.49% (SEM 0.17% range 0.2 to 1.13%); recently infected symptomatic adults 0.28% (SEM 0.11% range, 0.03 to 0.91%); and asymptomatic adults mean, 0.15% (SEM 0.06% range, 0.03 to 0.37%) (*p* < 0.05) Lower mean rotavirus-specific CD4 IFN-γ T-cell frequency 0.02% (SEM 0.007% range −0.01 to 0.07%) in infected children than in exposed adults 0.1% (SEM 0.02% range, 0.02 to 0.19%); symptomatically infected adults mean 0.18% (SEM 0.10% range 0.02 to 0.94%) and asymptomatic rotavirus infected adults mean 0.05%; SEM 0.01%; range, 0.01 to 0.09%) (*p* < 0.01). CD4 IL-13 T-cell frequency mean 0.02%; SEM, 0.009%; range, 0 to 0.06% detected in children but not adults but no predominance in CD4 IFN-γ or IL-13 T-cells in children.	Lower circulating frequency of Th1 and cytotoxic T-cells in infected children than adults Mixed Th1 and Th2 responses in children contrasted to predominantly Th1 in adults.
Makela et al., 2004 [26]	Healthy (T1D at risk) children: (*n* = 20); Healthy rotavirus exposed adults (*n* = 16)	Lymphoproliferation	Adults had stronger T-cell proliferation to bovine rotavirus (NCD) (*p =* 0.0001–0.0067), human rotavirus lysate (*p =* 0.0008–0.011) and purified human rotavirus (*p =* 0.0044–0.083) than any age group of children. Similar T-cell proliferation to PPD in children and adults (*p =* 0.53–0.91)	Children have weaker T-cell responses to rotavirus compared to adults. Rotavirus is a poor inducer of T-cells in comparison to mycobacterial tuberculin
Makela et al., 2006 [25]	Healthy children (T1D at risk, *n* = 183)	Lymphoproliferation	Children had a higher median T-cell proliferative response to TT and PPD than to purified rotavirus, human rotavirus lysate, or bovine rotavirus (NCD)	Rotavirus is a poor inducer of T-cells in comparison to mycobacterial tuberculin and tetanus toxoid
Parra et al., 2014 [30]	Healthy children (*n* = 5) and healthy adults (*n* = 25)	Cytokine secreting PBMC. Th1 CD4 and cytotoxic CD8 T-cells. CD4 and CD8 proliferation.	IFN-γ, TNF-α, GM-CSF, RANTES, MCP-1 and IL-10 secreting PBMC in adults but not children Lower frequencies of IFN-γ, TNF-α, and IL-2 CD4 T-cells against rotavirus than against TT (*p* = 0.0313) or Influenza (*p* = 0.0313) in both children and adults. Monofunctional (single IFN-γ or TNF-α secreting) rotavirus specific CD4 T-cells predominant in both adults and children	Diminished Th1 responses in children than adults. Rotavirus is a poor inducer of T-cells in comparison to tetanus toxoid and Influenza CD4 T-cell response to rotavirus involves predominantly Th1 subset
Rojas et al., 2003 [32]	RV-AGE children (*n* = 9); Healthy adults (*n* = 7)	Frequencies of Th1 and Th2 CD4 and cytotoxic CD8 T-cells	Both IFN-γ CD4 (*p* = 0.046) and IFN-γ CD8 (*p* = 0.028) T-cells against rotavirus detected in adults but only IFN-γ CD8 *p* = 0.018) and not CD4 T-cells (*p = 0.17*). detected in children with diarrhoea. Low but insignificant frequency of IL-4 CD4 T-cells against rotavirus detected in both adults and children (*p =* 0.15).	IFN-γ cytotoxic CD8 T-cells may be the main effector in acute rotavirus infected children Th2 CD4 T-cells may have a less significant role against rotavirus

Abbreviations: CD3 = cluster of differentiation 3. CD4 = cluster of differentiation 4. αβCD4 = alpha beta cluster of differentiation 4. CD8 = cluster of differentiation 8. αβCD8 = alpha beta cluster of differentiation 8. GM-CSF = granulocyte-macrophage colony-stimulating factor. IFN-γ = interferon gamma. IL-2 = interleukin 2. IL-10 = interleukin 10. IL=13 = interleukin 13. IL-17 = interleukin 17. mRNA = messenger ribonucleic acid. MCP1 = monocyte chemoattractant protein 1. NCD = Nebraska Calf Diarrhoea. OKT3 = anti-CD3 monoclonal antibody. OKT4 = anti-CD4 monoclonal antibody. OKT8 = anti-CD8 monoclonal antibody. PBMC = peripheral blood mononuclear cells. *p* = probability value. PPD = tuberculin purified protein derivative. RANTES = regulated on activation, normal T-cell expressed and secreted. RV-AGE = rotavirus acute gastroenteritis. SEB = staphylococcal enterotoxin B. SEM = standard error of measurement. T1D = type 1 diabetes. Th1 = T-helper type 1. Th2 = T-helper type 2. Th17 = T-helper type 17. TT = tetanus toxoid. TNF-α = tumor necrosis factor alpha. VP6-7 = viral protein 6-7.

**Table 5:** T-cell activation, proinflammatory, regulatory and homing phenotypes in response to rotavirus.

Author, Year [Ref]	Child Population	T-Cell Response	Finding	Interpretation
Dong et al., 2015 [21]	RV-AGE (*n* = 102); Healthy (*n* = 30)	Th17 and Tregs frequency	Frequencies of CD4^+^IL-17^+^Th17 cells and circulating IL-17 and IL-6 proinflammatory cytokines were increased (*p* < 0.05) in RV-AGE than healthy children. (*p* < 0.05) In contrast, the frequency of CD4^+^CD25^+^ Treg cells and levels of circulating IL-10 and TGF-β regulatory cytokines in children with rotavirus enteritis was significantly decreased when compared with the healthy children (*p* < 0.05).	Th17 cells play a role in the protective immune response to rotavirus CD4^+^CD25^+^ T-cells and regulatory cytokines lowered in rotavirus infection
Iwasa et al., 2008 [23]	Infant with acute rotavirus gastroenteritis (*n* = 1)	Th1 CD4 and cytotoxic CD8 T-cell frequencies	Elevated IFN-γCD4^+^ (14.85%) and CD8^+^ (77.58%) T-cell frequency during acute stage that decreased one month later to 3.46% and 0.19% respectively	IFN-γ Th1 CD4 and cytotoxic CD8 T-cells are effectors against acute rotavirus
Makela et al., 2006 [25]	Healthy (T1D at risk), *n* = 183)	IFN-γ, IL-4, IL-10 and TGF-β mRNA expression and T-cell proliferation	Positive correlation between PBMC IFN-γ, IL-4 and IL-10 mRNA secretion and lymphoproliferation against rotavirus (*r* = 0.48, *p* = 0.003, r = 0.46, *p* = 0.004, and *r* = 0.36, *p* = 0.026 respectively). No correlation with TGF-β	Rotavirus T-cell responses includes Th1 and Th2 effectors IL-10 and not TGF-β regulatory T-cells may be important immune regulators of the proinflammatory response
Wang et al., 2007 [34]	RV-AGE (*n* = 10); Healthy (*n* = 8)	Gene expression of T-cell immune markers	Elevated gene expression of inflammatory immune markers TNF- α, proIL-1β, IL-1 β, IL-6, IL-8, GRO- β, IL-1R antagonist, IFN- α/β receptor and IFN α/β -stimulated proteins in rotavirus infected children than healthy children Elevated CD4 T-cell activation CD4/CD69 (from 2.7% to 10.5% [mean, 5.5%]), CD4/CD83 (from 10.5% to 25.8% [mean, 16.6%]), and CD8 T-cell activation CD8/CD69 (from 1.6% to 8.3% (mean, 3.5%), CD8/CD83 (from 4.4% to 16.1% [mean, 7.8%]) in RV-AGE than in healthy children range 0% to 0.5% (mean, 0.3%) for CD4/CD69, from 0.1% to 4.0% (mean, 1.2%) for CD4/CD83, from 0.1% to 0.7% (mean, 0.3%) for CD8/CD69, and from 0% to 0.4% (mean, 0.2%) for CD8/CD83 respectively.	Rotavirus induces a pro-inflammatory immune response CD69 and CD83 activated CD4 and CD8 T-cells contribute to antiviral activity and recovery from disease in children
Mesa et al., 2010 [27]	RV-AGE (*n* = 53)	CD4^+^CD25^+^, CD4^+^CD25^+^CD127^low^, CD4^+^CD25^+^CD127^low^ TGF-β^+^ and CD45RA^+^ regulatory T-cells (Tregs) and IFN-γ producing CD4 T-cells	Rotavirus IFN-γ CD4 T-cells not affected by TGF-β regulation in children but in adults No difference in CD4^+^CD25^+^, CD4^+^CD25^+^CD127^low^ and CD4^+^CD25^+^CD127^low^ TGF-β^+^ Tregs in RV-AGE and non-RV-AGE Most CD4^+^CD25^+^CD127^low^ Treg cells and CD4^+^CD25^+^CD127^low+^TGF-β^+^ Treg cells in children are naïve phenotype (CD45RA)	TGF-β does not regulate IFN-γ^+^CD4-T cell to rotavirus in children. The naïve Treg profiles in children could result in their reduced immunomodulatory effects in response to rotavirus infection
Parra et al., 2014 [31]	Vaccine (*n* = 3)	CD62L^−^CD45RA^+/−^ and CD26L^+^CD45RA^−^ CD4 T-cells α4β7 and CCR9	Most of the rotavirus antigen VP6-7 tetramer^+^ experienced CD4 T-cells expressed α4β7, or expressed both, α4β7 and	Majority rotavirus CD4 T-cells are gut homing Generation of these T-cell gut homing phenotypes may be important for clearing rotavirus infection and protecting against re-infection
Rojas et al., 2003 [32]	RV-AGE (*n* = 9)	Frequencies of CD4 and CD8 T-cells producing IL-4 and IFN-γ	Detectable INF-γ CD8 (*p* = 0.018) but not INF-γ CD4 (*p =* 0.17) T-cells. IL-4 CD4 and IL-4 CD8 also not detected (*p =* 0.15).	INF-γ^+^ cytotoxic CD8 T-cells may be more important for initial clearance of infection than the CD4 T-cell subset
Rott et al., 1997 [33]	convalescing RV-AGE (*n* = 1)	T-cell proliferation	α4β7^hi^ blood lymphocytes showed a 2.6-fold greater proliferative response to rotavirus than α4β7^−^ cells (SI 4.07 versus 1.54 respectively)	Majority of rotavirus T-cell have α4β7^hi^ phenotype

Abbreviations: α4β7 = alpha 4 beta 7. CCR9 = C-C motif chemokine receptor 9. CD4 = cluster of differentiation 4. CD8 = cluster of differentiation 8. CD25 = cluster of differentiation 25. CD45RA = cluster of differentiation 45RA. CD62L = cluster of differentiation 62L. CD69 = cluster of differentiation 69. CD83 = cluster of differentiation 83. CD127 = cluster of differentiation 127. GRO-β = growth-regulated oncogene. IFN-α = interferon alpha. IFN- β = interferon beta. IFN-γ = interferon gamma. IL-1β = interleukin 1β. IL-1R = interleukin 1R. pro-IL-1β = precursor interleukin 1β. IL-4 = interleukin 4. IL-6 = interleukin 6. IL-8 = interleukin 8. IL-10 = interleukin 10. IL-17 = interleukin 17. mRNA = messenger ribonucleic acid. *p* = probability value. PBMC = peripheral blood mononuclear cells. *r* = Pearson’s correlation coefficient. RV-AGE = rotavirus acute gastroenteritis. Th1 = T-helper type 1. Th2 = T-helper type 2. Th17 = T-helper type 17. T1D = type 1 diabetes. TNF-α = tumor necrosis factor alpha. Treg = regulatory T-cell. TGF-β = transforming growth factor beta.

## Data Availability

Data is included within the article.

## Supplementary Materials

The following are available online at https://www.mdpi.com/article/10.3390/v14030459/s1, Table S1: PRISMA checklist, File S1: Search strategy example, Table S2: Quality assessment tool.
