# Supplementary material for: T-Cell Responses after Rotavirus Infection or Vaccination in Children: A Systematic Review
Source: Viruses. 2022 Feb 23;14(3):459. doi: 10.3390/v14030459 (PMC8951614; doi:10.3390/v14030459)
Supplement: Supplementary file 1 [file viruses-14-00459-s001.zip › File S1. Search strategy example.pdf]

File S1 Search strategy example

Database: Ovid MEDLINE(R) <1946 to February Week 4 2020>
Search Strategy:
----

1 exp T-Lymphocytes/ (322411)

2 ((thym* adj3 lymphocyt*) or (thym* adj3 cell*) or t-cell* or tcell* or t-lymphocyt* or tlymphocyt*).mp. (465331)

3 ((cd4-positive* adj3 cell*) or (cd4-positive* adj3 lymphocyte*) or (cd4-positive* adj3 t-cell*) or (cd4-positive* adj3 tcell*) or (cd4-positive* adj3 t-lymphocyt*) or (cd4-positive* adj3 tlymphocyt*) or (t4 adj3 cell*) or (t4 adj3 lymphocyt*) or (cd4+ adj3 cell*) or (cd4+ adj3 lymphocyt*)).mp. (122090)

4 ((helper adj3 cell*) or (helper adj3 lymphocyt*) or (t-helper adj3 cell*) or (t-helper adj3 lymphocyt*) or (helper-inducer adj3 t-cell*) or (helper-inducer adj3 tcell*) or (helper-inducer adj3 t-lymphocyt*) or (helper-inducer adj3 tlymphocyt*) or (helper adj3 t-cell*) or (helper adj3 tcell*) or (helper adj3 t-lymphocyt*) or (helper adj3 tlymphocyt*) or (inducer adj3 cell*) or (inducer adj3 lymphocyt*)).mp. (36567)

5 ((th-1 adj3 cell*) or (th1 adj3 cell*) or (th-1 adj3 lymphocyt*) or (th1 adj3 lymphocyt*) or (t-helper adj3 type-1) or (thelper adj3 type-1) or (type-1 adj3 t-cell*) or (type-1 adj3 tcell*) or (type-1 adj3 t-lymphocyt*) or (type-1 adj3 tlymphocyt*)).mp. (29030)

6 ((th-2 adj3 cell*) or (th2 adj3 cell*) or (th-2 adj3 lymphocyt*) or (th2 adj3 lymphocyt*) or (t-helper adj3 type-2) or (thelper adj3 type-2) or (type-2 adj3 t-cell*) or (type-2 adj3 tcell*) or (type-2 adj3 t-lymphocyt*) or (type-2 adj3 tlymphocyt*)).mp. (23362)

7 ((th-17 adj3 cell*) or (th17 adj3 cell*) or (th-17 adj3 lymphocyt*) or (th17 adj3 lymphocyt*) or (t-helper adj3 type-17) or (thelper adj3 type-17) or (type-17 adj3 t-cell*) or (type-17 adj3 tcell*) or (type-17 adj3 t-lymphocyt*) or (type-17 adj3 tlymphocyt*)).mp. (11110)

8 ((suppressor adj3 t-cell*) or (suppressor adj3 tcell*) or (suppressor adj3 t-lymphocyt*) or (suppressor adj3 tlymphocyt*) or (regulatory adj3 t-cell*) or (regulatory adj3 tcell*) or (regulatory adj3 t-lymphocyt*) or (regulatory adj3 tlymphocyt*) or (th3 adj3 cell*) or (th3 adj3 lymphocyt*) or (tr1 adj3 cell*) or (tr1 adj3 lymphocyt*) or (treg adj3 cell*) or (treg adj3 lymphocyt*)).mp. (44525)

9 ((cd8-positive* adj3 cell*) or (cd8-positive* adj3 lymphocyt*) or (cd8-positive* adj3 t-cell*) or (cd8-positive* adj3 tcell*) or (cd8-positive* adj3 t-lymphocyt*) or (cd8-positive* adj3 tlymphocyt*) or (t8 adj3 cell*) or (t8 adj3 lymphocyt*) or (cd8+ adj3 cell*) or (cd8+ adj3 lymphocyt*)).mp. (71907)

10 ((killer adj3 t-cell*) or (killer adj3 tcell*) or (killer adj3 t-lymphocyt*) or (killer adj3 tlymphocyt*) or (nkt adj3 cell*) or (nkt adj3 lymphocyt*) or (inkt adj3 cell*) or (inkt adj3 lymphocyt*)).mp. (10220)

11 ((epitheli* adj3 t-cell*) or (epitheli* adj3 tcell*) or (epitheli* adj3 t-lymphocyt*) or (epitheli* adj3 tlymphocyt*) or (intra-epitheli* adj3 t-cell*) or (intra-epitheli* adj3 tcell*) or (intraepitheli* adj3 t-cell*) or (intraepitheli* adj3 tcell*) or (intra-epitheli* adj3 t-lymphocyt*) or (intra-epitheli* adj3 tlymphocyt*) or (intraepitheli* adj3 t-lymphocyt*) or (intraepitheli* adj3 tlympocyt*) or (iel adj3 cell*) or (mucosa* adj3 t-cell*) or (mucosa* adj3 tcell*) or (mucosa* adj3 t-lymphocyt*) or (mucosa* adj3 tlymphocyt*) or (gamma-delta adj3 t-cell*) or (gamma-delta adj3 tcell*) or (gamma-delta adj3 t-lymphocyt*) or (gamma-delta adj3 tlymphocyt*)).mp. (10397)

12 ((mait adj3 cell*) or (mait adj3 lymphocyt*) or (mucosal-associated adj3 t-cell*) or (mucosal-associated adj3 tcell*) or (mucosal-associated adj3 t-lymphocyt*) or (mucosal-associated adj3 tlymphocyt*)).mp. (419)

13 ((cytotox* adj3 t-cell*) or (cytotox* adj3 tcell*) or (cytotox* adj3 t-lymphocyt*) or (cytotox* adj3 tlymphocyt*) or (lympholy* adj3 cell*) or (cell-mediated adj3 lympholy*) or lympholy* or (tc1 adj3 cell*) or (tc1 adj3 lymphocyt*) or (tc2 adj3 cell*) or (tc2 adj3 lymphocyt*)).mp. (46608)

14 1 or 2 or 3 or 4 or 5 or 6 or 7 or 8 or 9 or 10 or 11 or 12 or 13 (518662)

15 exp Antigens, Differentiation, T-Lymphocyte/ (73399)

16 ((t-cell* adj3 different*) or (tcell* adj3 different*) or (t-lymphocyt* adj3 different*) or (tlymphocyt* adj3 different*) or (t-cell adj3 antigen*) or (tcell adj3 antigen*) or (t-lymphocyt* adj3 antigen*) or (tlymphocyt* adj3 antigen*)).mp. (74182)
17 ((cd4 adj3 antigen*) or (cd4 adj3 molecul*) or (cd4 adj3 receptor*) or (cd4 adj3 protein*) or (cd4 adj3 glycoprotein*) or (t4 adj3 antigen*) or (t4 adj3 molecul*) or (t4 adj3 receptor*) or (t4 adj3 protein*) or (t4 adj3 glycoprotein*) or (leu-3 adj3 antigen*) or (leu-3 adj3 molecule*) or (leu-3 adj3 receptor*) or (leu-3 adj3 protein*) or (leu-3 adj3 glycoprotein*)).mp. (23004)
18 ((cd8 adj3 antigen*) or (cd8 adj3 molecul*) or (cd8 adj3 receptor*) or (cd8 adj3 protein*) or (cd8 adj3 glycoprotein*) or (t8 adj3 antigen*) or (t8 adj3 molecul*) or (t8 adj3 receptor*) or (t8 adj3 protein*) or (t8 adj3 glycoprotein*) or (leu-2 adj3 antigen*) or (leu-2 adj3 molecule*) or (leu-2 adj3 receptor*) or (leu-2 adj3 protein*) or (leu-2 adj3 glycoprotein*)).mp. (13824)
19 ((t3 adj3 antigen*) or (t3 adj3 molecul*) or (t3 adj3 receptor*) or (t3 adj3 protein*) or (t3 adj3 glycoprotein*) or (t3 adj3 complex*) or (cd3 adj3 antigen*) or (cd3 adj3 molecul*) or (cd3 adj3 receptor*) or (cd3 adj3 protein*) or (cd3 adj3 glycoprotein*) or (cd3 adj3 complex*) or (leu-4 adj3 antigen*) or (leu-4 adj3 molecule*) or (leu-4 adj3 receptor*) or (leu-4 adj3 protein*) or (leu-4 adj3 glycoprotein*)).mp. (17501)
20 15 or 16 or 17 or 18 or 19 (136030)
21 exp Lymphocyte Count/ (39023)
22 ((cd4 adj3 count*) or (cd4+ adj3 count*) or (t4 adj3 count*) or (cd4 adj3 number*) or (cd4+ adj3 number*) or (t4 adj3 number*) or (leu-3 adj3 count*) or (leu-3 adj3 number*)).mp. (39676)
23 ((cd8 adj3 count*) or (cd8+ adj3 count*) or (t8 adj3 count*) or (cd8 adj3 number*) or (cd8+ adj3 number*) or (t8 adj3 number*) or (leu-2 adj3 count*) or (leu-2 adj3 number*)).mp. (4782)
24 ((cd4-cd8 adj3 ratio*) or (t4-t8 adj3 ratio*)).mp. (8108)
25 21 or 22 or 23 or 24 (60291)
26 exp Th1-Th2 Balance/ (940)
27 ((th1-th2 adj3 ratio*) or (th1-th2 adj3 balance*)).mp. (2847)
28 26 or 27 (2847)
29 exp Receptors, Antigen, T-Cell/ (38190)
30 ((t-cell* adj3 receptor*) or (tcell* adj3 receptor*) or (t-lymphocyt* adj3 receptor*) or (tlymphocyt* adj3 receptor*) or tcr).mp. (63057)
31 (gamma-delta adj3 tcr).mp. (1465)
32 (alpha-beta adj3 tcr).mp. (2145)
33 29 or 30 or 31 or 32 (64680)
34 exp Immunity, Cellular/ (165487)
35 ((cell-mediated adj3 immunit*) or (cell* adj3 respons*) or (cell* adj3 immunit*) or (t-cell* adj3 immunit*) or (tcell* adj3 immunit*) or (t-lymphocyt* adj3 immunit*) or (tlymphocyt* adj3 immunit*)).mp. (260891)
36 34 or 35 (350015)
37 14 or 20 or 25 or 28 or 33 or 36 (750569)
38 Rotavirus Vaccines/ (2264)
39 ((rotavirus* adj3 vaccin*) or (rotavirus* adj3 immuni#ation) or rotarix or (rv1 adj3 vaccin*) or rotateq or (rv5 adj3 vaccin*) or rotavac or (rotavirus* adj3 116e) or (rotavirus* adj3 rix4414) or rotashield or rotasiil or (lanzhou adj3 rotavirus*) or (rotavirus* adj3 LLR) or rotavin-m1).mp. (3606)
40 38 or 39 (3606)
41 Rotavirus/ (8656)
42 (rotavirus* or (human* adj3 rotavirus*) or (rotavirus* adj3 antigen*) or (rotavirus* adj3 VP*) or (rotavirus adj3 NSP*) or (rotavirus* adj3 protein*) or (rotavirus* adj3 peptide*) or (rotavirus* adj3 particle*) or (rotavirus* adj3 pathogen*)).mp. (14416)
43 41 or 42 (14416)
44 Rotavirus Infections/ (7658)

45 ((rotavirus* adj3 infect*) or (rotavirus* adj3 enteri*) or (rotavirus* adj3 gastroenteri*) or (rotavirus* adj3 diarrh?ea) or (rotavirus* adj3 disease*) or (rotavirus* adj3 morbid*) or (rotavirus* adj3 mortal*)).mp. (9260)
46 44 or 45 (9260)
47 40 or 43 or 46 (14422)
48 37 and 47 (470)
49 limit 48 to (english language and yr="1973 -Current") (465)

****
