# Supplementary material for: T-Cell Responses after Rotavirus Infection or Vaccination in Children: A Systematic Review
Source: Viruses. 2022 Feb 23;14(3):459. doi: 10.3390/v14030459 (PMC8951614; doi:10.3390/v14030459)
Supplement: Supplementary file 1 [file viruses-14-00459-s001.zip › Table S2. Quality assessment tool.pdf]

Table S2. Quality assessment checklist for included individual studies

Domain	Quality appraisal checklist
**Introduction**	1. Was the research problem clearly described?
	2. Was the study well justified with rationale clearly stated?
	3. Were the research questions and/or /hypothesis stated?
	4. Were the research aims clearly stated?
**Method**	5. Was the study design clearly stated?
	6. Was the sample size provided?
	7. Was the sample size calculation including assumptions used clearly described?
	8. Was the study population clearly described?
	9. Were the study population inclusion and exclusion criteria clearly stated?
	10. Was the T-cell laboratory procedure well described?
	11. Was the statistical analysis well described?
	12. Were ethics procedures described?
**Results**	13. Was the participant flow clearly described?
	14. Were background characteristics of study population reported?

	15. Were the results linked to the research aim or methods?
**Discussion**	16. Was a summary of key findings stated?
	17. Was there a comparison and/or contrasting of findings to other relevant studies?
	18. Were strengths and limitations of the study considered and reported?
**Conclusion**	19. Were the conclusions logically based on the results?

**Domain**	**Q#**	Dong et. al 2015 (22)	Elaraby et al 1992 (23)	Iwasa et al 2008 (24)	Jaimes et al 2002 (25)	Makela et al 2006 (26)	Makela et al 2004 (27)	Mesa et al 2010 (28)	Offit et al 1992 (29)	Offit et al 1993 (30)	Parra et al 2014 (31)	Parra et al 2014 (32)	Rojas et al 2003 (33)	Rott et al 1997 (34)	Wang et al 2007 (35)	Weinberg et al 2018 (36)	Wood et al 1988 (37)	Yasukawa et al 1990 (38)	**% Score**
**Background**	1.	+	+	+	+	+	?	+	+	+	+	+	+	+	+	+	+	+	94
	2.	+	+	+	+	+	+	+	+	+	+	+	+	+	+	+	+	+	100
	3.	-	-	-	-	+	+	+	?	?	+	+	+	+	-	+	+	-	53
	4.	+	+	+	+	+	+	+	+	+	+	+	+	+	+	+	+	+	100

**Method**	5.	-	-	+	-	-	-	-	-	-	-	+	-	-	-	+	+	-	24
	6.	+	+	+	+	+	+	+	+	+	+	+	+	-	-	+	+	-	82
	7.	-	-	n/a	-	-	-	-	-	-	-	-	-	-	-	-	n/a	-	0
	8.	+	+	-	+	+	+	+	+	+	+	+	+	+	+	+	+	+	94
	9.	-	-	-	-	-	-	-	-	-	-	-	-	-	-	+	-	-	6
	10.	+	+	-	+	+	+	+	+	+	+	+	+	+	+	+	+	+	94
	11.	+	-	n/a	+	+	+	+	-	+	+	+	+	-	+	+	n/a	-	73
	12.	+	-	-	+	+	+	+	-	-	+	+	+	-	+	+	-	-	59
**Results**	13.	-	-	n/a	-	-	-	-	-	-	-	-	-	-	-	+	n/a	-	7
	14.	-	-	-	-	-	-	+	-	-	-	-	-	-	+	+	+	-	24
	15.	+	+	+	+	+	+	+	+	+	+	+	+	+	+	+	+	+	100
**Discussion**	16.	+	+	+	+	+	+	+	+	+	+	+	+	+	+	+	+	+	100
	17.	+	+	+	+	+	+	+	+	+	+	+	+	+	+	+	+	+	100
	18.	-	-	+	+	+	+	?	+	+	+	+	-	-	+	+	-	+	65
**Conclusion**	19.	+	+	+	+	+	+	+	+	+	+	+	+	+	+	+	+	+	100
**Overall score**		12	10	10	13	14	13	14	11	12	14	15	13	10	13	18	13	10	

**Total assessed**	19	19	16	19	19	19	19	19	19	19	19	19	19	19	19	16	19	
**% Score**	63	53	63	68	74	68	74	58	63	74	79	68	53	68	95	81	53	
**Quality**	**M**	**M**	**M**	**M**	**H**	**M**	**H**	**M**	**M**	**H**	**H**	**M**	**M**	**M**	**H**	**H**	**M**	

- n/a = not applicable; (+) = yes; (-) = no; (?) = unclear
- Quality threshold levels
  - 0% to 39% = low quality (L)
  - 40% to 69% = moderate quality (M)
  - 70% to 100% = high quality (H)
